# Deeper Penetration of Erythrocytes into the Endothelial Glycocalyx Is Associated with Impaired Microvascular Perfusion

**DOI:** 10.1371/journal.pone.0096477

**Published:** 2014-05-09

**Authors:** Dae Hyun Lee, Martijn J. C. Dane, Bernard M. van den Berg, Margien G. S. Boels, Jurgen W. van Teeffelen, Renée de Mutsert, Martin den Heijer, Frits R. Rosendaal, Johan van der Vlag, Anton Jan van Zonneveld, Hans Vink, Ton J. Rabelink, for the NEO study group

**Affiliations:** 1 Department of Nephrology and Einthoven laboratory for Vascular Medicine, Leiden University Medical Center, Leiden, the Netherlands; 2 Department of Clinical Epidemiology, Leiden University Medical Center, Leiden, the Netherlands; 3 Department of Physiology and Glycocheck, Maastricht University Medical Center, Maastricht, the Netherlands; 4 Department of Nephrology, Nijmegen Centre for Molecular Life Sciences, Radboud University Nijmegen Medical Centre, Nijmegen, the Netherlands; University of Arizona, United States of America

## Abstract

Changes in endothelial glycocalyx are one of the earliest changes in development of cardiovascular disease. The endothelial glycocalyx is both an important biological modifier of interactions between flowing blood and the vessel wall, and a determinant of organ perfusion. We hypothesize that deeper penetration of erythrocytes into the glycocalyx is associated with reduced microvascular perfusion. The population-based prospective cohort study (the Netherlands Epidemiology of Obesity [NEO] study) includes 6,673 middle-aged individuals (oversampling of overweight and obese individuals). Within this cohort, we have imaged the sublingual microvasculature of 915 participants using sidestream darkfield (SDF) imaging together with a recently developed automated acquisition and analysis approach. Presence of RBC (as a marker of microvascular perfusion) and perfused boundary region (PBR), a marker for endothelial glycocalyx barrier properties for RBC accessibility, were assessed in vessels between 5 and 25 µm RBC column width. A wide range of variability in PBR measurements, with a mean PBR of 2.14 µm (range: 1.43–2.86 µm), was observed. Linear regression analysis showed a marked association between PBR and microvascular perfusion, reflected by RBC filling percentage (regression coefficient β: −0.034; 95% confidence interval: −0.037 to −0.031). We conclude that microvascular beds with a thick (“healthy”) glycocalyx (low PBR), reflects efficient perfusion of the microvascular bed. In contrast, a thin (“risk”) glycocalyx (high PBR) is associated with a less efficient and defective microvascular perfusion.

## Introduction

Cardiovascular disease is the leading cause of death in developed countries and one of the earliest changes in the pathogenesis of cardiovascular disease is microvascular dysfunction [1]. Within the inner vessel wall, a luminal endothelial glycocalyx is strategically located to continuously interact with the flowing blood. This endothelial glycocalyx is a thick gel-like meshwork of proteoglycans, glycosaminoglycans and plasma proteins; it functions as an important biological modifier in the interaction between the blood and the vessel wall [2], [3]. Degradation and modification of the endothelial glycocalyx is, therefore, thought to be one of the earliest changes occurring in the pathogenesis of vascular disease [4], [5]. For example, the endothelial glycocalyx exerts an anti-inflammatory and anti-thrombotic role by covering various glycoprotein adhesion receptors for leukocytes [6] and platelets [7]. Also, the endothelial glycocalyx has a protective role against protein leakage, as shown by our group previously, when selective degradation of endothelial glycocalyx with hyaluronidase led to glomerular albumin leakage [4].

Another function of the endothelial glycocalyx has been proposed to be the regulation of microvascular perfusion. The concept that the glycocalyx contributes to the regulation of microvascular perfusion was originally hypothesized by the group of Duling in 1990 when they showed that the adenosine-induced increase in capillary tube hematocrit in hamster cremaster muscle vessels was diminished after enzymatic glycocalyx degradation [8]. Further evidence for the role of the glycocalyx in regulation of functional microvascular perfusion has subsequently been gathered [9]–[13]. Changes in glycocalyx composition have been demonstrated to result in a decrease of shear- dependent nitric oxide (NO) -mediated arteriolar vasodilation [14], to decrease functional capillary density [15] and to induce platelet- and leukocyte adhesion in microvessels [6], [7], [16]; all effects that potentially affect microvascular perfusion. Loss of microvascular perfusion is a principal process in chronic organ failure, including heart, kidney and vascular dementia. The central concept is that endothelial (EC) activation turns pericytes into myofibroblasts, resulting in loss of capillaries, tissue hypoxia and subsequent organ fibrosis. However, there is currently no data on the relation between health of the endothelial glycocalyx and microvascular perfusion regulation in man.

Of the several methods to measure glycocalyx health *in vivo*, we have utilized the property of the endothelial glycocalyx to function as a barrier to maintain a certain distance between red blood cells (RBC) and the endothelial cell membrane [17]–[19]. To determine the spatio-temporal variation in radial displacement of individual RBCs into the endothelial glycocalyx in microvessels we used sidestream darkfield (SDF) imaging. SDF imaging is a non-invasive technique which visualizes hemoglobin within the RBC by reflected light emitting diode (LED) light from the microvasculature [20]. With this technique, it has been shown that the RBCs (diameter of 7 to 8 µm) maintain a certain distance from the endothelium of capillaries (diameter of 5 µm), which was postulated to be due to the barrier function of the glycocalyx against the RBC [19]. Using SDF imaging, we imaged the longitudinal and radial distribution of RBC in sublingual microvessels to obtain information about the *in vivo* endothelial glycocalyx barrier properties in humans [21]. This concept has recently been tested in various patient groups with cardiovascular disease or risk factors, such as end-stage renal disease [17], [21], stroke [18], premature coronary artery disease [22] and critically ill patients (septic and non-septic)[23], in which it was indicated that a perturbed glycocalyx allowed the erythrocytes to penetrate deeper towards the endothelium, resulting in an increase in the perfused boundary region (PBR). In addition to the lateral RBC movements, the longitudinal presence of RBCs (along known as vascular segments per surface area) is measured, allowing a simultaneous examination of the glycocalyx exclusion properties and the microvascular spatio-temporal RBC perfusion. We hypothesized that impaired glycocalyx barrier properties in the sublingual microcirculation is associated with changes in microvascular perfusion capacity in the general population.

## Methods

### Study Design and study population

We performed a cross-sectional analysis among participants recruited for the Netherlands Epidemiology of Obesity (NEO) study [24] to examine the association between endothelial glycocalyx integrity and microvascular perfusion. The NEO study is a population-based, prospective cohort study of 6,673 individuals aged between 45 and 65 years, with oversampling of overweight and obese individuals, to study pathways that lead to obesity-related diseases for a better understanding of the mechanisms underlying development of disease in obesity.

Detailed information about the study design and data collection has been described previously [24]. Men and women aged between 45 and 65 years with a self-reported BMI of 27 kg/m^2^ or higher and living in the greater area of Leiden (in the west of the Netherlands) were eligible to participate in the NEO study. In addition, all inhabitants aged between 45 and 65 years from one municipality (Leiderdorp, the Netherlands) were invited, irrespective of their BMI, allowing for a reference BMI distribution. SDF imaging was performed in a random subgroup of 937 participants from the 1394 participants who were included from January 2012 to October 2012. The study was carried out in accordance with the Declaration of Helsinki. Approval was obtained from the Committee of Medical Ethics of Leiden University Medical Center and all participants gave written informed consent.

### General Measurements

Prior to the NEO study visit, participants completed a questionnaire about demography, lifestyle and medical history and fasted for at least 10 hours. Participants came to the research site in the morning to undergo several baseline measurements including anthropometric measurements, and blood sampling. Hemoglobin (Hb) was measured by the SLS hemoglobin detection method and hematocrit (Hct) by the RBC cumulative pulse height detection method with a Sysmex XE-2100 system (Sysmex, UK).

Extensive physical examination was conducted, including anthropometry and blood pressure measurements. Blood pressure was measured three times in a seated position on the right arm with a 5 minutes resting interval using a validated automatic oscillometric device (OMRON, Model M10-IT, Omron Health Care Inc., IL, USA). Prehypertension and hypertension were diagnosed according to JNC 7 criteria [25].

### Imaging of microcirculation

**Figure 1:**
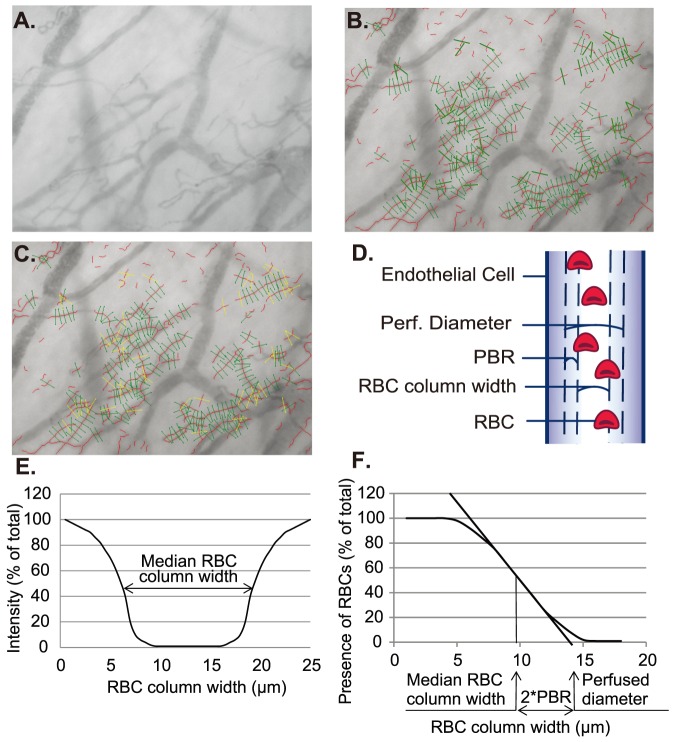
Glycocheck algorithm on endothelial PBR determination and microvascular perfusion properties. **A**) Red blood cells (RBC) are detected through reflection of light emitting diodes by hemoglobin. Images captured by the sidestream darkfield camera are sent to the computer for quality checks and assessment. The black contrast is the perfused lumen of the vessels. **B**) In each recording, the software automatically places the vascular segments (green), every 10 µm along the vascular segments (black contrast). **C**) After the acquisition, for the analysis, the software undergoes several quality check in the first frame of each recording (see text), to select vascular segments with sufficient quality for further analysis. Invalid vascular segments (yellow) are distinguished from the valid vascular segments (green). During the whole recording session of 40 frames, the percentage of time in which a particular valid vascular segment has RBCs present is used to calculate RBC filling percentage. **D**) Depiction of the concept of glycocalyx thickness by lateral RBC movement is shown here. **E**) For each vascular segment, the intensity profile is calculated to derive median RBC column width. **F**) Then, the distribution of RBC column width is used to calculate the perfused diameter, median RBC column width, and subsequently the perfused boundary region (PBR).

Intravital microscopy was performed at bedside with a SDF camera (MicroVision Medical Inc., Wallingford, PA) to visualize the sublingual microvasculature. The SDF camera uses green light emitting diodes (540 nm) to detect the hemoglobin of passing RBC. The images were captured using a 5× objective with a 0.2 NA (numerical aperture), providing a 325- fold magnification in 720×576 pixels (tissue dimension: 950×700 µm; tissue area: 0.665 mm^2^) at 23 frames per second. The system makes up to 840 measurements of the width of the RBC column at a specific site. These individual width measurements are obtained after 8-fold pixel size interpolations of the corresponding radial intensity profiles, allowing detection of small changes in width between individual width measurements as small as 0.1 µm, which translates in accuracy of estimates of boundary layer of approximately 0.05 µm. The image acquisition is automatically mediated through the Glycocheck software (Glycocheck BV, Maastricht, the Netherlands) (Figure 1. for illustration of software algorithm). It detects the dynamic lateral RBC movement into the glycocalyx, which is expressed as the perfused boundary region (PBR, in µm). Therefore, a perturbed or degraded glycocalyx would allow more RBCs to penetrate deeper towards the endothelial surface, which is reflected by an increase in PBR (figure 1d.). The method of calculating the PBR has been used and validated in various publications [17], [18], [21]–[23].

In short, the software automatically identifies all available measurable micro-vessels (below 30 µm thickness) during the acquisition by contrast between the RBC and the background, in focus and without movement of the imaging unit (raw data in figure 1a). With this, it places vascular segments every 10 µm along the length of these vessels. Subsequently, a sequence of 40 frames is recorded containing, on average, 300 major vascular segments (depicted as green lines in figure 1b). Next, the observer moves the camera to a different location to record another 40 frames, until a minimum total of 3,000 vascular segments are placed. After acquisition, the software undergoes a series of quality checks to only measure vascular segments that are of good quality. First, on the first frame of each recording session, ten line markers are placed every 0.5 µm along each side of the vascular segments (total of 21 line markers). Only those that have sufficient contrast on more than 60% of the 21 line markers of each vascular segment are considered a valid vascular segment (green lines in figure 1c), in contrast to the invalid vascular segment (yellow lines in figure 1c). Since all vascular segments are placed every 10 µm, the length of perfused microvascular length can be expressed by multiplying 10 µm and the number of valid vascular segments per area of tissue visualized (valid microvascular density). For the second quality check, during the measurement of RBC column widths for all 40 frames of recording session, the software screens for minimal RBC width, position of RBC column and the signal-to-noise ratio. At this step, the software calculates the percentage of vascular segments with RBC present during all 40 frames of the recording session, to determine the RBC filling percentage. Both the valid microvascular density and the RBC filling percentage were used as an estimate for the microvascular perfusion. For the last quality check, the curve fit for calculating the median RBC column widths is tested also for irregularities (figure 1e). Thus, after all these quality checks, those that fulfill these criteria are subject to analysis for the radial distribution of RBC, as a measurement of glycocalyx function.

For each vascular segment, the dynamic lateral position of RBCs (per RBC column width) is determined. The intensity profiles of the distribution of RBC column widths are used to calculate a cumulative distribution. From this distribution, the median RBC column width is obtained while a linear regression analysis of the RBC column width is performed to derive the perfused diameter (the X-axis intercept of the extrapolated regression line) is calculated (figure 1f). Then, the PBR is defined as the distance between RBC column width and perfused diameter and is calculated using the equation, ([Perfused diameter – median RBC column width]/2). Next, the calculated PBR values, classified according to their corresponding RBC column width between 5–25 µm, are averaged to provide a single PBR value for each participant.

### Statistical Analysis

In the NEO study there is an oversampling of persons with a BMI of 27 kg/m^2^ or higher. For the present study, to correctly represent associations in the general population [26], adjustments for the oversampling of individuals with a BMI ≥27 kg/m^2^ were made. This was done by weighting individuals towards the BMI distribution of participants from the Leiderdorp municipality, whose BMI distribution was similar to the BMI distribution of the general Dutch population [26]. All results were based on weighted analyses. Consequently, results apply to a population-based study without an oversampling of BMI ≥27 kg/m^2^.

Participants with unsuccessful measurements through SDF imaging (including participants with incomplete recording sessions (less than 10; n = 16) and abnormal video images, such as consistent presence of air bubbles (n = 6), all PBR outlier values), preventing correct analysis were excluded (n = 22). In the end, 915 participants were included in the analysis of data. Baseline characteristics of the population were expressed as mean (±standard deviation [SD]), median (interquartile range), or as percentage. We performed linear regression analysis to investigate the associations of perfused boundary region with RBC filling percentage and valid microvascular density, while adjusting for age and sex. All statistical analyses were performed using SPSS version 20.0 (Chicago, IL), STATA Statistical Software (Statacorp, College Station, Texas) and GraphPad version 5.0 (GraphPad Prism Software Inc., San Diego, CA). A p-value <0.05 was defined as statistically significant.

## Results

### General clinical characteristics

The clinical characteristics of individuals with measured SDF are shown in Table 1. All participants were between 45 to 65 year old. Of all participants, 42% were lean, 42% were overweight, and 16.0% were obese. The mean systolic blood pressure was 131 mmHg (S.D.: 16 mmHg) and diastolic blood pressure was 83 mmHg (S.D.: 10 mmHg), with 36% with hypertension.

**Table 1:** General Clinical Characteristics.

	Total (n = 915)
**Gender (% of male/female)**	46/54
**Age (year)**	56.1±6.0
**BMI (kg/m^2^)**	26.4±4.2
**Hb (mmol/L)**	8.71±1.1
**Hct**	0.41±0.12
**Hypertension (%)**	33.5%
**Systolic BP (mmHg)**	131.0±16.2
**Diastolic BP (mmHg)**	83.2±9.7

Hb, hemoglobin; Hct, hematocrit; BP, blood pressure. Data is presented as mean ± standard deviation.

### Determination of microcirculatory properties

From the RBC column width and perfused diameter, perfused boundary region was calculated to derive the glycocalyx barrier function (Table 2, #1 to #3). Overall, the mean PBR was 2.14 µm with a wide range of variability (range: 1.43–2.86 µm) (table 2). Despite the wide distribution of PBR in our cohort, there was a difference in PBR between men and women (mean: 2.09 µm and 2.18 µm, respectively).

**Table 2:** Sidestream darkfield imaging derived variables characteristics.

#	Parameter	Total	Men	Women
1	RBC Column width (µm)	10.56±1.12	10.74±1.10	10.41±1.11
2	Perfused diameter (µm)	14.84±1.18	14.92±1.19	14.77±1.17
3	Perfused boundary region (µm)	2.14±0.25	2.09±0.24	2.18±0.25
4	Total microvascular density (µm/mm^2^)	4314±882	4223±821	4391±924
5	Valid microvascular density (µm/mm^2^)	3213±691	3144±627	3272±736
6	RBC filling (%)	73.2±5.0	74.0±4.8	72.4±5.0

Data is presented as mean ± standard deviation.

In addition to the glycocalyx barrier function by means of PBR, microvascular perfusion outcomes were calculated, resulting in a mean valid microvascular density of 3213 µm/mm^2^ (table 2, #5) and a mean RBC filling percentage of 73.2% (table 2, #6). In other words, there is 3246 µm of perfused microvessels per mm^2^ of tissue area which was in 73.2% positive for the presence of RBCs at a given time point during the 40 frames of video recording.

### Association of the glycocalyx barrier properties with microcirculatory perfusion

**Figure 2:**
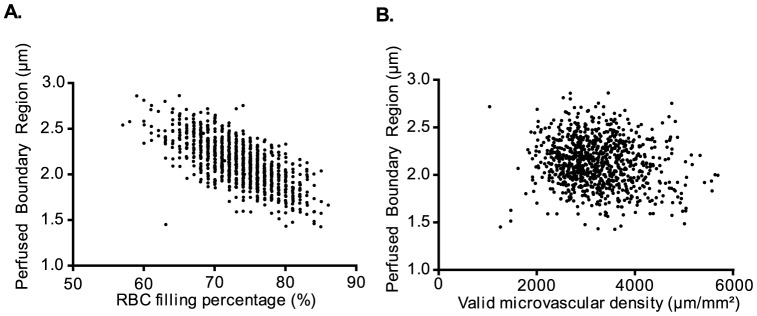
Scatterplot between PBR and outcomes of microvascular perfusion. The perfused boundary region (PBR), a measurement for glycocalyx accessibility to red blood cells (RBC), is associated significantly with spatio-temporal aspects of microvascular perfusion variables: **A**) RBC filling percentage (percentage of time in which a particular vascular segment is perfused) **B**) Valid microvascular density. In particular, lower PBR (less accessible glycocalyx, thus a better and thicker glycocalyx) is associated with higher RBC filling percentage (temporal perfusion).

To test the hypothesis whether impaired glycocalyx barrier properties are associated with impaired microvascular perfusion, we examined the association between PBR and the two used estimates for microvascular perfusion (Table 3 and figure 2). Of the two microvascular perfusion outcomes, RBC filling percentage was associated with PBR (regression coefficient β: −0.034; 95% CI: −0.037 to −0.031) and explained 47% of variability of perfused boundary region (R^2^: 0.475, p<0.001). The valid microvascular density was also associated with PBR but explained the variability of PBR to a lesser extent (R^2^: 0.030), although still significant. Examples of video-clips obtained from participants with different PBR values and corresponding changes in microvascular perfusion are shown in Movie S1 and S2 (high and low PBR, respectively).

**Table 3:** Linear regression analysis show association between perfused boundary region and microvascular perfusion parameters.

Independent variable*	Regression coefficient β	R-Square	95% CI for coefficient β†	
			Lower Bound	Upper Bound
RBC filling percentage	−0.034	0.475	−0.037	−0.031
+Age, sex, BMI‡	−0.034	0.482	−0.036	−0.031
Valid microvascular density§	−0.054	0.02	−0.083	−0.025
+Age, sex, BMI‡	−0.059	0.06	−0.087	−0.030

*Dependent variable: Perfused boundary region (PBR).

† 95% confidence interval for regression coefficient β.

‡ Linear regression analysis adjusted for age, sex and BMI.

§ Valid microvascular density expressed as millimeter of microvessel length per mm^2^ of area of tissue (mm/mm^2^) for linear regression analysis due to difference in scale from PBR.

## Discussion

We have measured the RBC accessibility into the endothelial glycocalyx, i.e. perfused boundary region (PBR) together with RBC filling percentage and valid microvascular density in the sublingual microvasculature of over 900 participants recruited for the NEO study.

In this study we observed a strong association between changes in endothelial glycocalyx properties (PBR) and estimates of microvascular perfusion in the sublingual microcirculation (Figure 2 and Table 3). The strongest association existed between PBR and RBC filling percentage, which represents the microvascular perfusion changes over time.

**Figure 3:**
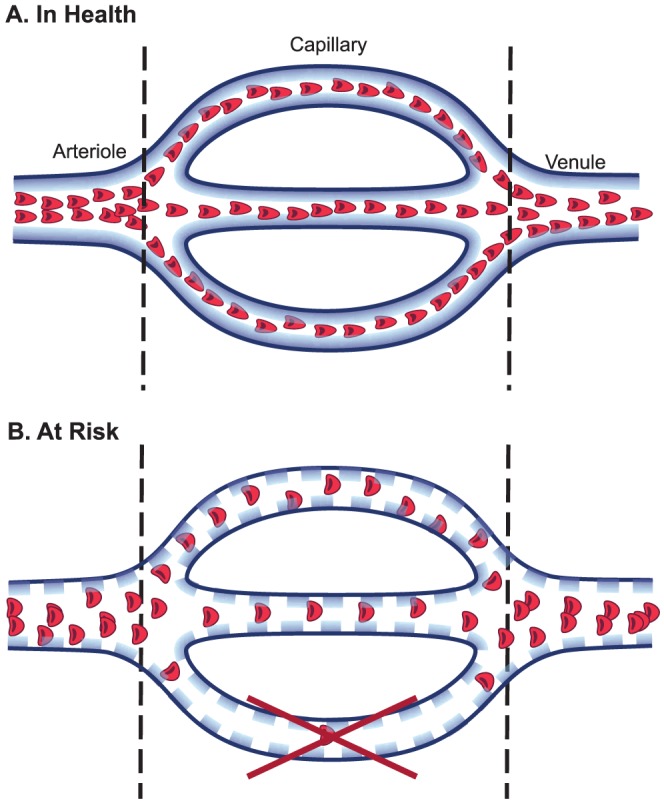
Schematic illustration on relation between glycocalyx accessibility and microvascular perfusion regulation. **A**) Healthy state: Intact glycocalyx prevents red blood cells (RBC, red dots) from penetrating into its domain, reflected by a low perfused boundary region (PBR), and nicely aligned elongated RBC. The vessels are well perfused (higher tube hematocrit of microvessel and elongated shape of erythrocyte) resulting in a higher percentage of vessel segments with RBC present at any particular time point (high RBC filling percentage). **B**) Risk State: Altered composition of glycocalyx (lined dots) allows RBCs to penetrate deeper into the glycocalyx, closer to the anatomical border of lumen (endothelium), reflected by the high PBR. Due to the widening of RBC distribution width and volume, there is more space in between each RBC, as shown by decreased RBC filling percentage (less positive contrast per vascular segment per time point). Also, prolonged state of glycocalyx degradation leads to edematous and non-functioning vessels, leading to shorter vessel density per area of tissue (reduced valid microvascular density in risk PBR), depicted by the loss of bottom vessel.

The mechanically stable gel-like layer on the endothelial surface limits the area in which RBCs and large plasma proteins are distributed (RBC exclusion properties of glycocalyx), which is reflected by a low PBR (figure 3a). An earlier observation of individual RBC in capillaries showed that compression of the RBCs by intact glycocalyx result in more elongated RBCs (7–8 µm length and 3–4 µm width), and therefore a more efficient and faster passage of these RBCs [19]. Also, this would lead to increased RBC longitudinal passage, allowing more oxygen exchange capacity. The relation between PBR and vascular perfusion has been studied, however, in a variety of different physiological stimulants, such as by exercise, nitroglycerine, adenosine or insulin [9], [10], [22]. For instance, nitroglycerine challenge in a healthy population led to an increase in PBR and a recruitment of reserve capacity, further arguing that in a healthy state of acute physiological need, the glycocalyx has the potential to become more porous to facilitate the increased metabolic demand [10]. With such transient stimuli, both PBR and perfusion increases, leading to a temporary local increase in oxygen/nutrient exchange. Therefore the observed variation in PBR and its correlation with perfusion could theoretically be explained by this dynamic regulation. Nonetheless, we have performed our measurements in a strictly controlled environment (e.g. overnight fasting, resting for more than 15 minutes, exclusion of caffeine and smoking). Thus, the observed variation in PBR and clear negative correlation (figure 2b) with the microvascular perfusion would argue for differences in vascular health in our population.

Therefore, in an unhealthy vascular state, PBR increase (reflecting increased outward penetration of RBC) together with decreased RBC filling percentage and functional microvascular density, reflects poor perfusion (figure3b). When the glycocalyx is degraded for a prolonged time, protein extravasation increases with subsequent edema formation [4], [27], and a reduction in nitric oxide bioavailability [14], all together leading to endothelial dysfunction and perfusion defects [10], [15]. For instance, in patients with diabetes, there is increased hyaluronidase activity [28], which probably decreases glycocalyx volume and functional capillary density [15], [28], [29]. The increase in distance between RBCs (reflected by low RBC filling percentage) might be due to both RBC changes into a wider shape [19] and more intravascular volume in which RBC could distribute. Ultimately, these changes lead to slow, diffuse and inefficient perfusion [30], as schematically depicted in figure 3.

Variability in the width of the RBC column, and thus PBR could theoretically also decrease to lower values when the glycocalyx layer becomes very thin. However, it is empirically and consistently found in all available and known (clinical) data, that PBR progressively increases with the severity of disease and glycocalyx damage. The most striking increases in PBR are found in critically diseased patients with severe septic shock in the ICU (unpublished data), who are supposed to have shed off the glycocalyx [6], [23]. In these subjects the level of PBR increase correlates directly with mortality and cardiovascular organ complications. It therefore appears that relatively infrequent deeper penetration of RBCs into the layer during degradation/damage of glycocalyx precedes the radial outside movement of the majority of the RBCs in the RBC column, resulting in increases in PBR on top of potential increases in median width of the RBC column. Moreover, in the present study, we performed the measurements in a large group of relative healthy participants, making the loss of glycocalyx in the microvasculature also a relative unlikely event.

A question obviously is, whether glycocalyx assessment of the sublingual microcirculation is representative for glycocalyx dimensions in other tissues. Glycocalyx thickness and functional properties may indeed differ substantially from one organ to another [31]. However, as with other measurements of endothelial function, systemic factors will affect representative functional endothelial changes throughout the circulation. For example, using the SDF technique we and others have shown that sublingual vascular changes correlate with changes in the microvasculature of gastric, renal and nervous system organs [17], [18], [21], [32]–[38]. In addition, we have shown in diabetic subjects that glycocalyx dimensions correlated with systemic glycocalyx volume changes [28]. Also, changes in the sublingual PBR in patients with coronary artery disease or stroke imply that local microvascular abnormalities indeed reflect changes in the systemic microvasculature [18], [22].

During the past few years, there have been several methodological advances introduced in estimating glycocalyx dimensions. In the past, SDF glycocalyx dimensions were calculated as the difference in RBC column width before (functionally perfused capillary diameter) and after (anatomical capillary diameter) leukocyte passage [28], [39]. Thereby, it measures the transient change in thickness due to leukocyte- induced compression of the glycocalyx. These previous measurements have shown reduced glycocalyx dimensions in various cardiovascular diseases such as diabetes [28], [29], obesity [39], and hypercholesterolemia [40]. However, it is a labor-intensive process by manually capturing leukocyte passages for a longer time period and analyzing the results. In addition, it is subject to investigator bias. The current method of SDF imaging we have used, which detects the dynamic changes in the erythrocyte movements, acquires and analyses the recordings in an unbiased, fully automated fashion.

While this technique estimates the glycocalyx thickness and microvascular perfusion non-invasively and automatically, it does have its limitations. First, the SDF camera only detects RBCs. Therefore, the perfused diameter and PBR is not the anatomical diameter of the vessel or the thickness of the anatomical glycocalyx, respectively. Nonetheless, by measuring the distribution of RBCs in a dense spatio-temporal manner, the software measures the location of the RBCs and can thereby statistically calculate the perfused diameter and PBR. In addition, we have recently validated that the measurement for PBR reflects actual anatomical glycocalyx damage, as shown by reduced distance between endothelial cell and RBC after hyaluronidase treatment using intravital microscopy [21]. Second, by visualizing the spatio-temporal location of the RBCs, the software estimates the microvascular perfusion by how often a certain vascular segment has a passing RBC. However, conventional outcome of perfusion variables includes RBC velocity or the anatomical microvascular density in a tissue [32]. Also, during the acquisition of approximately 2 seconds, the software cannot detect the vessel if there are no RBCs passing through. Therefore, it cannot visualize all vessels.

In conclusion, SDF imaging of the mucosal microvasculature in an adjusted subgroup of participants recruited for the NEO study, to represent associations in a general population, showed a wide range of variability in RBC accessibility to the glycocalyx, which was negatively associated with microvascular perfusion. This means that with a thick (“healthy”) glycocalyx (reflected by a low PBR) there is efficient perfusion of the microvascular bed. In contrast, a thin (“risk”) glycocalyx (reflected by a high PBR) is associated with less efficient and perturbed perfusion. The strong, and until now unknown, correlations of PBR with microvascular perfusion in man, indicate that values of PBR might allow us to report on relations between these parameters during follow up of these subjects, and relate it to future cardiovascular risk. This is clinically very relevant as microvascular rarefaction is a principal process in development of chronic organ failure.

## Supporting Information

Movie S1 **Video clip of participants with high perfused boundary region with altered microvascular perfusion.** A single recording session (from ten or more recording sessions per measurement) is extracted from random participants with high and low (Movie S2) PBR to illustrate the difference in level of perfusion. Video clip of a participant with high PBR (2.52 µm) shows altered microvascular perfusion. Descriptively, the movement of RBCs seem sluggish. Automated quantitative analysis through the software shows that the RBC filling percentage was 65.3% and the functional microvascular density (total length of perfused vessel per area of tissue) is 1985 µm/mm^2^.
(AVI)

Movie S2 **Video clip of participants with low perfused boundary region with normal microvascular perfusion.** Video clip of a participant with low PBR (1.84 µm) shows fast and efficient passage of RBCs through the microvessels. Quantitatively, the functional microvascular density is longer 6782 µm/mm^2^ compared to that of participant with high PBR (in Movie S1). The RBC filling percentage of this participant was higher at 74.1%, which means that the time in which a particular vascular segment contains an RBC present is increased. Note: While the functional microvascular density is based on the individual video clip, the PBR and RBC filling percentage are not just from the shown video clip, but calculated from all recording sessions.
(AVI)
